# Farmers’ perceptions on the causes of cassava root bitterness: A case of konzo-affected Mtwara region, Tanzania

**DOI:** 10.1371/journal.pone.0215527

**Published:** 2019-04-18

**Authors:** Matema L. E. Imakumbili, Ernest Semu, Johnson M. R. Semoka, Adebayo Abass, Geoffrey Mkamilo

**Affiliations:** 1 Department of Soils and Geological Sciences, Sokoine University of Agriculture, Morogoro, Tanzania; 2 The International Institute of Tropical Agriculture, Dar es Salaam, Tanzania; 3 Roots and Tubers Department, Naliendele Agricultural Research Institute, Mtwara, Tanzania; Feroze Gandhi Degree College, INDIA

## Abstract

In areas where konzo (a cassava cyanide related paralytic disorder) persists, the agronomic factors causing increased cyanogenic glucoside levels in cassava, during periods without water stress, are hardly known. However, through their assessment of cassava root toxicity, using its bitter taste, farmers may have noticed factors unrelated to water stress that additionally influence the cyanogenic glucoside content of cassava cultivated in these areas. Increased cassava root bitterness is often associated with an increase in cyanogenic glucoside levels, making it a good indicator of changes in root cyanogenic glucoside content. Bitter cassava varieties that are preferentially planted by people living in most konzo-affected areas, are an additional known contributor to high cyanogenic glucosides. It is water stress that further increases the inherent toxicity of the planted bitter cassava varieties. Using konzo-affected Mtwara region in Tanzania as a case study, a household survey was carried out to identify the overlooked agronomic factors that additionally influence cyanogenic glucoside levels in cassava cultivated in konzo-affected areas. A total of 120 farmers were interviewed and they mentioned a number of factors unrelated to water stress, as agronomic factors that influenced cassava root bitterness and hence cyanogenic glucoside production in cassava. The mentioned factors included; certain soil characteristics (14.2%), plant age at harvest (7.5%), poor weeding (0.8%), piecemeal harvesting (0.8%), and branch pruning (0.8%). The revealed factors constitute permanent environmental characteristics and crop management practices commonly used by farmers living in konzo-affected Mtwara region in Tanzania. The revealed factors could be contributing to increased cyanogenic glucoside levels in cassava, during periods without water stress in areas where konzo persists.

## Introduction

Cassava (*Manihot esculenta* Crantz) is one of the world’s most important food crops. Despite its importance, cassava unfortunately contains potentially toxic cyanogenic glucosides, which release poisonous hydrogen cyanide upon hydrolysis. Without access to foods containing sulphur amino acids, continuous ingestion of cyanogenic glucosides from improperly processed cassava products, often results in cases of cassava cyanide intoxication [1]. Rural poor cassava dependent communities are particularly at risk. Cases of cassava cyanide intoxication have been reported in a number of countries in sub-Saharan Africa, such as, the Democratic Republic of Congo (DRC), Mozambique, Tanzania, Cameroon, Central African Republic and Angola [2–6]. The reports consisted of cases of acute cyanide intoxication, but more commonly of a cassava cyanide health disorder called konzo (spastic paraparesis), which results in an irreversible paralysis of legs [7–10].

One reason given for the high intake of cassava cyanide that results in konzo epidemics, is increased cyanogenic glucoside levels in fresh cassava roots and in cassava products produced from them [4,11]. Researchers mainly attribute the increase in cyanogenic glucoside levels to water stress from prolonged droughts that coincide with most epidemics of konzo [4,12] and also to the planting of bitter cassava varieties, which are preferred by many farmers [4,13]. The naturally high cyanogenic glucoside content of bitter cassava varieties are further increased by water stress. Dry season (inter-seasonal dry spells) water stress, is similarly known to result in increased cyanogenic glucoside levels in cassava [3,14,15]. During the dry season, cassava cyanogen levels can increase by 9–10 times their normal levels [16].

Small epidemics and sporadic cases of konzo have been concurrently observed in some communities, creating near persistent exposures to konzo [5,17]. Konzo is persistent in some rural areas of Mozambique, DRC and Tanzania [6]. In areas where konzo persists, high cyanogenic glucoside levels in cassava may also occur outside periods of water stress [17]. This suggests that factors other than droughts and dry seasons could also be contributing to increased cyanogenic glucoside levels in cassava, bringing about persistent cassava cyanide intoxication and konzo, in these areas. Other agronomic factors that are characteristic of cassava cropping systems in konzo-affected areas, could thus be additionally influencing cassava cyanogenic glucoside production.

Farmers in Africa generally use the bitter taste of cassava roots to perceive the potential toxicity of cassava [13,18,19]. Research has shown that a greater portion of cassava varieties perceived as bitter and toxic by farmers, do indeed contain higher cyanogenic glucoside levels than cassava varieties perceived as being sweet [18]. However, this may not always be the case, as cassava contains other bitter compounds [20], making validation necessary. However, during a period in which a konzo epidemic was experienced, families had been reported to complain that cassava roots were more bitter than normal during that period [21]. Thus, through taste perceptions, most people had become conscious of an increase in cassava root bitterness that led to cases of cassava cyanide intoxication. This report shows that the bitter taste of cassava roots can be used to determine increased root toxicity. Cassava root taste can also be used to assess changes in the degree of bitterness in roots of a particular cassava variety [22,23].

When sporadic cases of konzo occur un-related to water stress, it is more difficult to explain the agronomic factors leading to increased cyanogenic glucoside levels in cassava. However, being able to observe the crop throughout the year, farmers may have knowledge of other agronomic factors influencing cassava root bitterness and consequently root cyanogenic glucoside levels. This study was hence carried out to investigate the agronomic factors that lead to increased cassava root bitterness and consequently increased cyanogenic glucoside levels in cassava, during periods without water stress according to the perception of farmers. Konzo-affected Mtwara region in Tanzania was used as a case study.

## Materials and methods

### Description of the study area

Three districts have been reportedly affected by cassava cyanide poisoning in Mtwara region, namely Masasi, Mtwara rural and Newala districts [4,7,24]. This study, however, focused on villages of Mtwara rural and Newala districts. The two districts covered in the survey are two of the five districts found in Mtwara region (S 10^o^16'25", E 40^o^10'58"). Soils in Mtwara region and in the two districts (Mtwara rural and Newala) have a low natural soil fertility [25,26]. They are predominantly sandy and have been classified as Ferralic Cambisols [25,27]. The study areas mainly lie in Tanzania’s coastal lowlands agroecological zone (C2) [25,27]. The rainfall in the Mtwara region is unimodal and ranges from 800 to1000 mm/year and the maximum and minimum temperatures vary from 29 to 31 ^o^C and from 19 to 23 ^o^C, respectively [25]. Mtwara region experiences long dry seasons. The wet season is only 3 to 4 ½ months long with unreliable rainfall onset dates [25]. The wet season is further shortened by the occurrence of a characteristic 3 to 4 weeks intra-seasonal dry period, which usually occurs in February [25,28].

### Field methods and tools

The study was a household survey in which structured interviews were individually administered to farmers to collect information on their perceptions on the agronomic causes of cassava root bitterness. Both closed and open ended questions, were used to collect this information (S1 Text). During the interviews, farmers were asked what caused cassava roots to become bitter, while the plant was still growing in the field. Farmers were additionally asked to explain their responses. For instance, if farmers mentioned that increased bitterness was as a result of the soil, then they were asked to provide details on the soil characteristics or types that resulted in the increase of cassava root bitterness. If the mentioned factor only affected a few varieties or all cassava varieties then the farmers had to also provide this information. Visits were also made to crop fields to observe how the farmers practiced cassava cultivation. Questions on cassava cropping practices were asked to better understand the growing conditions of cassava. Farmers had also been asked to describe the soils on their crop field. The study had been conducted in October 2014, during the main cassava harvest period in Mtwara region. The questionnaire had been pre-tested and adjusted accordingly before conducting the actual survey.

### Participant sampling

Household heads (either the wife or husband, if both spouses were present) from Mtwara rural district and Newala district, with full knowledge of the cropping history of their crop fields, were selected to be interviewed. The household heads (farmers) were picked from eight randomly selected konzo-affected villages. Four villages were selected from each district using simple random sampling [29]. The villages selected from Mtwara rural district were namely, Njengwa, Nyundo, Niyumba and Kiromba, while the villages Mdimba, Ngalu, Songambele and Mkunjo were selected from Newala district. The selected villages were among the 18 villages visited during a konzo rehabilitation and prevention program that was carried out from 2008 to 2009, by the Tanzania Food and Nutrition Centre (TFNC) and the Tanzania Red Cross Society (TRCS), with technical support from the Australian National University (ANU) [4].

Using simple random sampling and a 2012 census list for each village, 15 households were picked from each of the eight selected villages to participate in the survey. This had been done by first assigning a unique number to every household in a village in the order they appeared in the census list. The list of numbers representing the total number of households in a village were then randomised using the RANDBETWEEN function in Microsoft Excel [30]. The first 15 households that appeared on the randomised village list were then picked. Access to the census lists for each village, had been given by each respective village administrative committee, headed by a village headman.

Prior to the survey, an official letter acknowledging the research and consenting for it to proceed, had been obtained from the government administrative offices overseeing Mtwara region. After examining and understanding what was exactly involved in conducting the research, the regional government office made no reference for the need of obtaining ethical clearance from the Tanzania Commission for Science and Technology (COSTECH). COSTECH acts as the research review board in Tanzania. According to their understanding the research did not threaten to breach any ethics and was hence allowed to proceed. The letter from the regional government offices was then presented to the district administrative offices in Mtwara rural district and Newala district. After they had also consented for the research to proceed, short meetings were then held with members of each village administrative committee to give clarification on the study and to ask them to kindly participate in it. After understanding what was involved and seeing the importance of the research to them, the village administrative committees verbally consented for the research to proceed. Arrangements were then made to get verbal informed consent from the selected household heads to participate in the study. Verbal consent was obtained due to the low literacy levels of people living in these communities. Although some people could write, all participants were treated the same, to avoid making some of them uncomfortable.

Members of the village administrative committees, together with key government staff working in the villages, like agriculture camp officers, assisted in mobilising the farmers. Before getting participant consent in some villages, all selected participants had been brought together and the research explained to them by the principle researcher with help from staff from Naliendele Agricultural Research Institute (NARI). Where this was not possible, the people mobilising the households clarified the research to each selected household before getting their consent to participate in the study. A total of 59 and 61 household heads, were respectively selected from Mtwara rural district and Newala district, which brought the total number of selected household heads (farmers) to 120. The principal researcher had also carried a letter of introduction from their host institution to help identify them. The letter also indicated the principal researchers’ affiliation and the purpose of the study.

### Socio-economic characteristics of study participants

The study participants (farmers) were mostly male household heads (Table 1). The average age of the farmers was 50 years. The study sample had a higher number of older adult farmers (> 50 years old) with longer experience in cassava farming. The farmers had mainly only attained primary school education with most only going as far as grade four or five. The household sizes of these farming communities were mainly small and were on average composed of only five household members.

**Table 1:** Socio-demographic characteristics of study participants.

	Mtwara rural district	Newala district	Both districts
Socio-demographic factor	n = 59	n = 61	n = 120
	(%)	(%)	(%)
1. Sex			
Females	25.0	36.7	31.0
Males	75.0	63.3	69.0
2. Age groups (years)			
< 25	0.0	3.3	1.7
25–34	13.6	11.5	12.5
35–50	44.0	36.0	40.0
> 50	42.4	49.2	45.8
3. Education			
None	16.9	25.4	21.2
Primary (grades 1–7)	81.4	69.5	75.4
Ordinary secondary (grades 8–11)	1.7	5.1	3.4
Advanced secondary (grades 12–13)	0.0	0.0	0.0
Tertiary	0.0	0.0	0.0
4. Household size			
< 6	60.3	68.3	64.4
6–10	38.0	31.7	34.8
> 10	1.7	0.0	0.8

**Fig 1:**
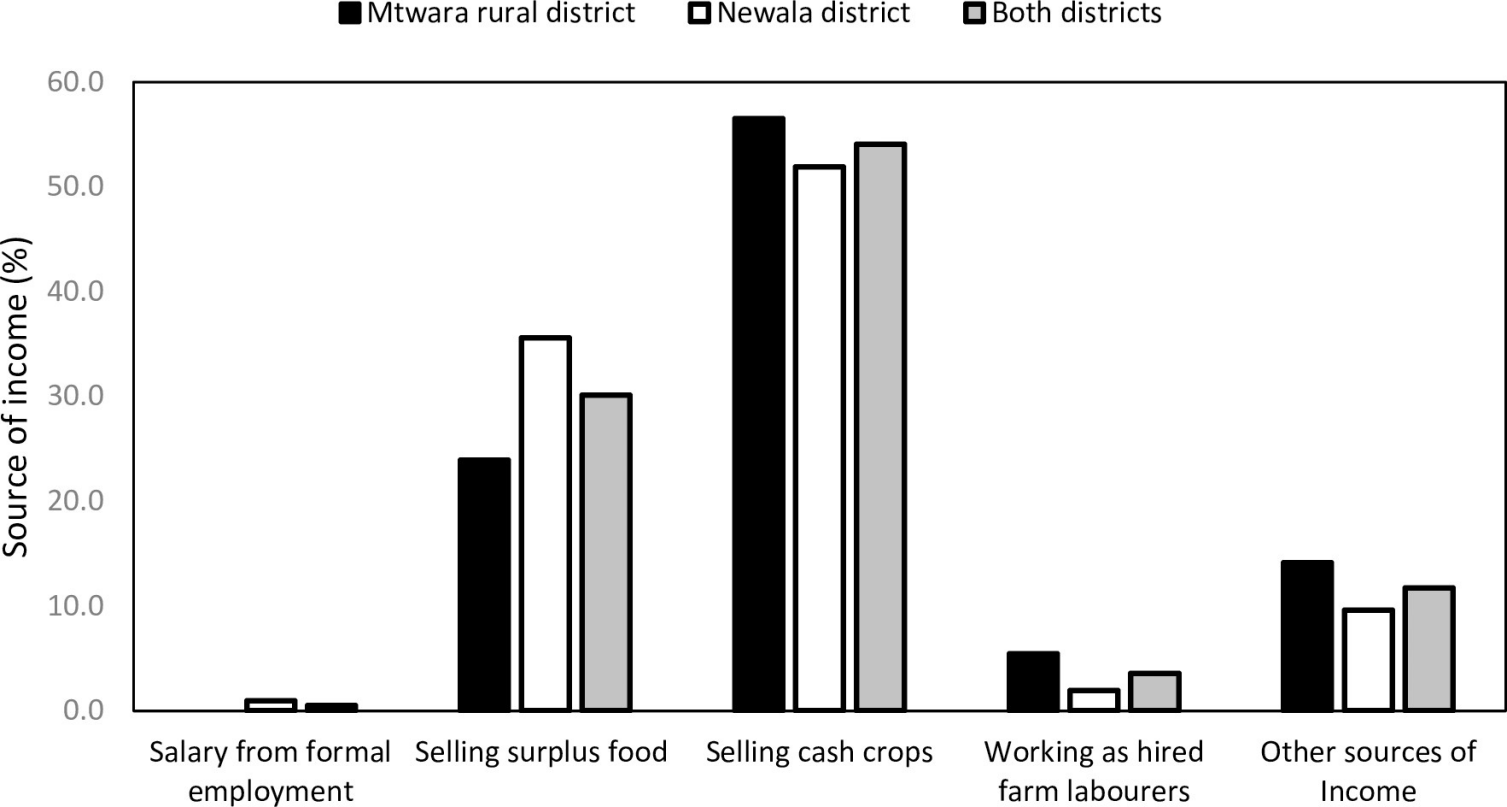
Main sources of income.

The farm households mainly got their income from selling cash crops and surplus food crops (Fig 1). The main cash crops were cashew nuts followed by cassava, if both districts are considered together. Households commonly sold cassava to other households within the same village. Some households, however also sold their cassava to buyers outside their village. Households were forced to buy cassava when they runout of their own stored supply of cassava. The importance of cassava as a food crop in these communities also made the crop an important readily available source of cash for households. Cassava was sold as a surplus food crop by 60.0% of the interviewed farmers in Mtwara district, while it was sold as a surplus food crop by only 20.6% of the farmers in Newala district. Maize (31.5%) was more prominent than cassava as a sold surplus food crop in Newala district. Maize was becoming prominent in Newala district due to government efforts to help farmers diversify their crop production in order to reduce their dependence on cassava. Although not so common, households also obtained income by selling animals, like chickens and goats. The selling of animals has been grouped in the category of other sources of income in Fig 1.

### Cassava cropping practices and characteristics of soils on crop fields

Most of the farmers planted cassava early in the wet season, immediately after the onset of the rains (Table 2). The wet season mainly starts in December and ends in April in Mtwara region. Early planting is hence around December and January and any planting later than January is considered late, particularly because of the dry spell that occurs in February. Some farmers in both districts however also practiced dry season planting. In this planting method, cassava stems were planted right after harvesting cassava. As the name suggests, dry season planting was carried out during the dry season, mainly in September and October.

**Table 2:** Cassava cropping practices and characteristics of soils on crop fields.

	Mtwara rural district	Newala district	Both districts
Cropping practices and soil characteristics	n = 59	n = 61	n = 120
	(%)	(%)	(%)
1. Common cassava planting times			
Early planting (December, January)	71.2	93.2	82.2
Late planting (February, March, April)	0.0	0.0	0.0
Dry-season planting (September, October)	28.8	6.8	17.8
2. Farmers that planted sweet cassava varieties	86.4	67.2	76.7
3. Farmers that planted bitter cassava varieties	91.5	93.4	92.5
4. Farmers that planted improved cassava varieties	1.7	1.6	1.7
5. Texture of soils on crop fields			
*Tifu*-*tifu* (loamy soil)	22.0	50.9	36.9
*Kichanga* (sandy soil)	76.0	45.3	60.2
*Mfinyanzi* (clayey soil)	2.0	3.8	2.9
6. Colour of soils on crop fields			
*Nyekundu* (red soil)	20.7	33.3	26.0
*Kijivu* (grey soil)	6.9	0.0	4.0
*Nyeusi* (black/dark soil)	13.8	38.1	24.0
*Nyeupe* (white soil)	58.6	28.6	46.0

The planting of both sweet and bitter cassava varieties was common amongst farmers (Table 2). Bitter cassava varieties were however planted in greater quantities, mainly because farmers were assured of harvesting something by planting them. Unlike sweet cassava varieties, bitter varieties could deter pests and thieves and they could also store longer when left unharvested in the field. Cassava flour produced from bitter cassava varieties was also preferred for making the staple food, *ugali*, which is a stiff porridge mainly consumed at lunch and dinner with vegetables and/or protein foods. Bitter cassava varieties were planted in a households’ main cropping field. This field was larger in size and located some distance away from the households’ home. On the other hand, sweet cassava varieties were mainly grown closer to the home, in home gardens, where they could be easily watched over. While it was more common to plant both sweet and bitter cassava varieties, a few farmers however preferred to only plant sweet or bitter cassava varieties alone. Almost all the cassava varieties planted were local cassava varieties. This might have been due to the low dissemination of improved cassava varieties to these areas.

Table 2 also shows the common textures and colours of soils on crop fields in konzo-affected areas of Mtwara region. The soil texture and colour names are all based on a local soil classification system used by the farmers. Most soils on crop fields were sandy and white coloured. White sandy soils were described as being single grained and as having poor fertility. Sandy soils were additionally described as soils that were easy to till but poor at retaining water. The farmers described red soils as soils that easily lost fertility and they added that red soils could not be continuously cropped for more than 3 years. Darkish sandy soils and loamy soils were described as being fertile, while clayey soils were described as hard soils with low fertility.

### Data analysis

The collected data was analysed as frequencies and frequency percentages (relative frequency multiplied by 100) [29], using GenStat package, Edition 14.

## Results and discussion

Table 3 shows the factors perceived by farmers as contributors of cassava root bitterness. The percentage of farmers that attributed root bitterness to each factor is also shown. The factors can be broadly categorised into genotype (variety), environment and crop management factors. It is important to note that while some factors were mentioned more than others, this does not mean that they are less significant contributors of cassava root bitter; this can only be proved by research.

**Table 3:** Farmers’ perceptions on factors causing cassava root bitterness.

Factors influencing root bitterness†	Mtwara rural district		Newala district		Both districts	
	‘Yes’ responses (n = 59)		‘Yes’ responses (n = 61)		‘Yes’ responses (n = 120)	
	number	(%)	number	(%)	number	(%)
1. Variety type	59	100.0	61	100.0	120	100.0
2. Environmental factors						
a. Soil characteristics	1	1.7	16	26.2	17	14.2
b.Seasonal changes	5	8.5	0	0.0	5	4.2
3. Farmers’ agronomic practices						
a.Plant age at harvest	0	0.0	9	14.8	9	7.5
b.Poor weeding	1	1.7	1	1.6	2	0.8
c.Piecemeal harvesting	1	1.7	0	0.0	1	0.8
d.Branch pruning	1	1.7	0	0.0	1	0.8

†Depending on its n value, each mentioned factor had 59, 61 or 120 chances of being mentioned in Mtwara rural district, Newala district and in both districts combined, respectively.

### Variety type

The type of variety was described by farmers as being the ultimate contributor of cassava root bitterness. They explained that bitter cassava varieties naturally produced bitter tasting roots, while sweet cassava varieties naturally produced roots that were sweet tasting. Researchers have found that variations in the cyanogenic glucoside content of varieties have a genetic basis [31]. Hence, in agreement with the farmers’, based on their respective genetic compositions, sweet cassava varieties are more likely to always produce sweet tasting roots and bitter cassava varieties, bitter tasting roots. The farmers were aware that some of the varieties they preferred to plant were very bitter and toxic, even if they continued to plant them. The bitter cassava varieties, *Salanga* and *Limbanga*, were most revered for their toxicity, as they were very bitter.

### Environmental factors

#### Soil characteristics

Soil characteristics were the second most perceived contributor of cassava root bitterness, after variety type (Table 3). Most responses on soils as a cause of cassava root bitterness were given by farmers from Newala district. This was probably because they were most affected by it. The variety *Kigoma* had been specifically identified as having the tendency of changing from sweet to bitter depending on a soils characteristics. Red clayey soils, fatigued soils, sandy soils and red soils were the four soil characteristics (or types of soils) associated with cassava root bitterness. Fatigued soils (43.8%) and red clayey soils (43.8%) were identified by most farmers as the major causes of cassava root bitterness. Farmers described fatigued soils as soils that had lost their fertility due to being continuously cultivated. They described red clayey soils as being red coloured soils that could be used for building and for making pottery. Fewer respondents mentioned red soils (6.2%) and sandy soils (6.2%). It is however not clear whether the red soils mentioned by some respondents are the same as the red clayey soils.

Farmers believed that red soils changed the normal taste of cassava roots to a bitter taste. Some farmers mentioned that red soils under anthills is what caused cassava to become bitter. The farmers gave special emphasis to the red clayey soils located in the Makote area of Newala district as always inducing a bitter taste in cassava roots. The red soils were described as having a thin ‘white’ surface soil layer (horizon), followed by a thick red coloured soil layer from which they got their descriptive name. The red colour of soils indicates the presence of high concentrations of nutrient poor iron oxides, which are products of intense weathering. The red soils on the Makonde plateau, where the two study districts are located, are described as impoverished and as having a poor nutrient holding capacity (cation exchange capacity (CEC) < 10 cmol/kg) [28]. The red soils mentioned by farmers can thus be concluded to be nutrient poor soils.

Some farmers complained that soils the on their crop fields easily became ‘tired’ or ‘fatigued’ after being consecutively cultivated for only a few years and that cultivating them in this state increased cassava root bitterness. They attributed the fatigue or exhaustion of soils on their crop fields to a loss soil fertility. In agreement with farmers soil fatigue is described as the exhaustion of soils due to the depletion of essential plant nutrients that leave soils nutrient poor. Hence, like the red soils, fatigued soils are also nutrient poor. Nutrient poor soils can cause nutrient stress in cassava and this can probably lead to increased root bitterness and hence increased cyanogenic glucoside levels in cassava.

A few studies have been carried out to demonstrate the effects of poor soil fertility on cassava root bitterness and on cyanogenic glucoside production in cassava roots. Some studies have shown that an improved supply of the nutrients nitrogen (N), phosphorus (P) and potassium (K) in soils, could help reduce total hydrogen cyanide levels (a measure of cyanogenic glucoside content) in cassava roots [23,32]. Reduced root bitterness of cassava has also been reported with an improved supply of N, P and K in soil [23]. These findings probably explain the perceived increase in cassava root bitterness on nutrient poor soils. Higher total cyanide levels in cassava roots, were however obtained in cassava grown on more fertile Fluvisols and Andosols, compared to levels found in cassava grown on highly weathered nutrient poor Nitisols [33]. This shows that nutrient poor soils may not always lead to increased cassava root bitterness. Increased cassava root bitterness was also observed on nutrient rich soils, with higher levels of basic cations and organic matter [34]. The pH, organic matter and K levels of the nutrient rich soils (pH as high as 7.8; organic matter as high as 8.2%; K as high as 1.9 cmol/kg) were however very high and unsuitable for optimal cassava growth [35]; this probably stressed the cassava plants and led to the observed increase in root bitterness.

Sandy soils had additionally been pointed out as a cause of cassava root bitterness. It is important to mention that due to their low CEC and lower moisture retention capacity, the problems associated with sandy soils arise both from poor fertility and poor moisture retention. The observed increase in cassava root bitterness on sandy soils could thus be due to both nutrient stress and water stress in this semi-arid region. However, like sandy soils, red clayey soils are also nutrient poor and also capable of causing water stress. Water stress arises as under low soil moisture conditions clay soils hold water more tightly, limiting its availability to plants. With clay soils as additional contributors of water stress, it can be seen that 50.0% of the soil types mentioned by farmers contributed to cassava root bitterness by them being able to worsen plant water stress.

#### Natural water stress conditions

A few farmers had observed that natural water stress conditions due to seasonal changes (dry and wet seasons) also influenced bitterness of cassava roots (Table 3). The five farmers that mentioned this were all from Mtwara rural district. The cassava varieties that had their root taste changed with season included the varieties, *Liwoyoka*, *Kigoma mafia*, *Musa Saidi*, *Nachinyanya*, *Badi*, *Vincenti*, *Mnalile Kuchumba* and *Mtukane*. All varieties mentioned, except for *Musa Saidi*, were sweet cassava varieties. Almost all the farmers mentioned that the bitter taste arose in the dry season. In agreement with the farmers observation, water stress caused by seasonal dry periods, is able to increase cyanogenic glucoside levels in cassava roots [36]. Furthermore, konzo, and thus high cyanogenic glucoside levels in cassava, also occurs in a seasonal pattern, with most cases occurring during the dry season [3,14]. One study found a strong association (r^2^ = 0.9) between normal yearly cyclic changes in precipitation and cassava cyanide intoxication that had resulted in konzo [15]. These findings further support the farmers’ observation of increased cassava root bitterness with seasonal changes, although not so obvious to most of them.

### Farmers’ agronomic practices

After soil type, cassava plant age at harvest or the length of time cassava roots were left unharvested, was the next most stated contributing factor to cassava root bitterness (Table 3). Although only mentioned by farmers in Newala district, farmers from both districts had a tendency of leaving cassava unharvested for long periods of time for the same reasons. The two sweet cassava varieties, *Kigoma* and *Kifuru*, were pointed out as being particularly prone to becoming bitter when left unharvested for long. Like the farmers observations, cyanogenic glucoside production in cassava is known to be dependent on growth stage (plant age) [37,38]. In one study, a reduction in cyanogen levels was observed with increased plant age (6, 8, 10 and 12 months after planting (MAP)) in flour produced from roots of a bitter cassava variety [39]. Conversely, no differences were observed in the cyanogen content of fresh cassava roots of a sweet cassava variety, harvested at three month intervals starting from 12 to 24 MAP [36]. Unlike the farmers’ observations, none of the research findings reported an increase in root bitterness with plant age. The farmers’ perceived increase in root bitterness with plant age, for sweet cassava varieties, may probably be due to a loss of cassava root quality in these varieties.

The farmers additionally believed that roots of late maturing bitter cassava varieties, when harvested ‘early’ (at 12–18 MAP), were immature and very toxic. They explained that cassava flour produced from roots of young bitter cassava varieties was toxic, even if it was produced using their more efficient traditional forms of cassava processing. In agreement with the farmers, young cyanogenic plants are known to contain higher amounts of cyanogenic glucosides [40]. Cassava is however normally mature by 12–18 MAP, even if the bitter cassava varieties were considered as immature at this plant age by the farmers. Leaving bitter cassava varieties to grow for a much longer time period, was a method used by the farmers to reduce cassava cyanide toxicity. Bitter cassava varieties were usually left unharvested until 24–36 MAP.

Weeds were also a concern to farmers as an additional factor contributing to cassava bitterness (Table 3). Some farmers explained that when cassava was poorly weeded, the roots it produced tended to be bitter. Weeds tend to grow faster and to seriously compete with cassava for light, water and nutrients, thus subjecting cassava plants to stress. Biotic stress factors, like weeds, are able to influence cyanogenic glucoside production in cassava plants and probably cassava root taste. In agreement with the farmers’ perception, delayed weeding or no weeding at maturity had been reported to result in increased cassava root bitterness in another study [34].

Another cause of cassava root bitterness was piecemeal harvesting (Table 3). Some farmers carried out piecemeal harvesting, although it was more common for them to harvest all the cassava in a field at once. Piecemeal harvesting is a traditional cassava harvesting method used to achieve longer storage of cassava roots by harvesting only needed roots at a time, while leaving the rest of the roots un-harvested and still attached to cassava plants in the field. Farmers observed that once the first roots had been removed from a cassava plant, the unharvested roots that were still attached to the plant eventually became bitter. It is however unclear whether increased root bitterness was observed immediately after harvest or after some days, weeks or months. Hardly any studies have been carried out to investigate the effects of piecemeal harvesting on cassava cyanogenic glucoside production. Piecemeal harvesting however introduces plant wounds. Under certain conditions, plant wounding can increase inherent plant toxins, like cyanogenic glucosides, in various plant species [41]. There is however hardly any data on the effects of wounding on cyanogenic glucoside accumulation in cassava.

**Fig 2:**
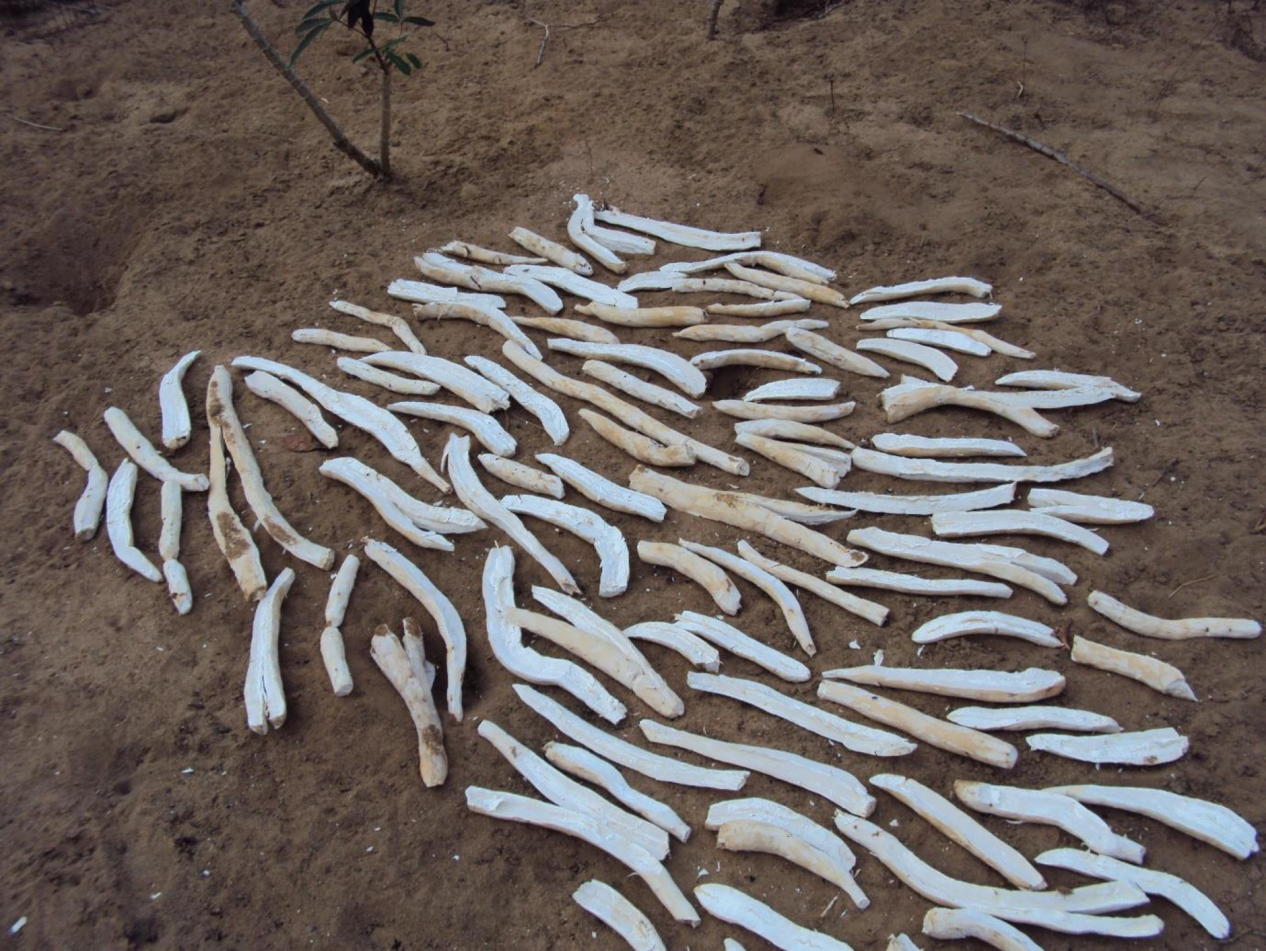
Cassava chips left to dry on bare ground.

Another reason for increased root bitterness arose from another common agronomic practice carried out by farmers in Mtwara region. The practice was branch pruning (debranching) of cassava plants (Table 3). Farmers’ cut-off cassava branches from plants during branch pruning. A considerable portion of the stem was left behind, as branches were the main parts targeted during the pruning process. It is important to note that branch pruning is carried out a bit differently from ratooning, which leaves behind only a stump of a cassava plant. Branch pruning was carried out to shorten cassava plants, making browsing easier for goats. Goats tended to damage tall cassava plants as they struggled to reach for the leaves. Branch pruning also allowed for faster regrowth of much needed cassava foliage. Cassava leaves were an important source of ruminant feed in these areas, in addition to them being an important source of vegetables for the people. Branch pruning was additionally used to obtain cassava branches for use as mats for drying cassava chips (locally called *makopa*) in the field. This occurred when farmers still needed their cassava plants to continue growing, like after piecemeal harvesting. Using cassava branches in this way avoided the direct placement of freshly peeled clean cassava chips on the bare ground when drying them (Fig 2). There could be other reasons for farmers branch pruning cassava plants, the mentioned reasons were just a few of the reasons obtained while conducting the study.

A few studies have looked at the effects of pruning on cyanogenic glucoside content in cassava roots. In one study, the effects of pruning showed no significant effects on the root cyanogen contents of six cassava varieties pruned at 9 MAP and harvested consecutively after 0, 2, 4, 6, 8, 10, 15, 20, 25, 28 and 39 days [42]. Only 30 cm of the cassava stem had been left on plants after pruning in this study. In another study, lower cyanogen levels were observed in various cassava varieties after pruning [43]. In the latter study, only leaves of cassava plants had been pruned, leaving behind the full branches and stems. Pruning had been carried out 7 days before harvest, at 12 MAP. The two studies had been carried out slightly differently and none of their findings explain the farmers’ observations of increased root bitterness with branch pruning. This is probably because the methods used do not exactly mimic how the farmers carried out the practice. The season in which pruning is carried out, the age at which plants are pruned, the time period between pruning and root harvest and how plants are pruned, all need to be taken into consideration before a reliable conclusion can be made. Another important point to note is that, like piecemeal harvesting, branch pruning, especially when it involves cutting off stems or branches also wounds cassava plants, hence inducing plant stress. As mentioned previously plant stress could lead to increased cyanogenic glucoside levels and probably to increased cassava root bitterness.

## Conclusions

As revealed by the perceptions of farmers, using cassava root taste, the agronomic factors that possibly contribute to increased cyanogenic glucoside levels in cassava during periods without water stress, include: certain soil characteristics (or types of soils) that induce nutrient stress and water stress, and agronomic practices used by farmers like, the age at which they harvest cassava, poor weeding practices, piecemeal harvesting and branch pruning. The mentioned factors could have contributed to the reported persistent episodes of konzo in Tanzania and could also be contributing to newly occurring cases of konzo in these areas, although not being reported. The revealed agronomic factors could also be responsible for causing increased cyanogenic glucoside levels outside periods of water stress in other areas where konzo persists. Research is however needed to validate the effects of the revealed agronomic factors on root cyanogenic glucoside production, in order to understand their significance.

## Supporting information

S1 Text Questionnaire. (DOCX)

S2 Text Questionnaire symbol key. (DOCX)

S1 Dataset Farmer perceptions. (XLSX)
