# Supplementary material for: Farmers’ perceptions on the causes of cassava root bitterness: A case of konzo-affected Mtwara region, Tanzania
Source: PLoS One. 2019 Apr 18;14(4):e0215527. doi: 10.1371/journal.pone.0215527 (PMC6472768; doi:10.1371/journal.pone.0215527)
Supplement: S1 Text — (DOCX) [file pone.0215527.s001.docx]

**QUESTIONNAIRE**

**Household information**

Village: ________________________________________ District: ___________________________

1. Age of person being interviewed? _________ years; or year of birth (**1 9** ___ ___)
2. Gender of person being interviewed? Male [ ] Female [ ]
3. Household size? ___________
4. Please **circle** the highest year of school completed:

| **None** | **Primary** | | | | | | | **Ordinary Secondary** | | | | **Advanced Secondary school** | | **Tertiary** |
| --- | --- | --- | --- | --- | --- | --- | --- | --- | --- | --- | --- | --- | --- | --- |
| 0 | 1 | 2 | 3 | 4 | 5 | 6 | 7 | 8 | 9 | 10 | 11 | 12 | 13 | 14+ |

1. **List the family’s main sources of income** and indicate order of importance where **1** is the most important and **7** the least important

| **Family depends on cash from** | **Tick** | **Specify jobs, business type or items sold** | **Order of importance** |
| --- | --- | --- | --- |
| 1. Salary from formal employment |  |  |  |
| 1. Sell of food surplus i.e. food grown for home consumption only sold when cash is needed |  |  |  |
| 1. Sell of crops grown specifically for sell i.e. cash crops |  |  |  |
| 1. Hired labour |  |  |  |
| 1. Income from cooperative farmer groups (SACCOS) |  |  |  |
| 1. Small trade |  |  |  |
| 1. Other: |  |  |  |

**Perceptions on the agronomic causes of increased bitterness of cassava roots**

1. What causes cassava roots to become bitter, while a plant is still in the field? (Don’t read out the suggested responses)

1 = Type of variety [ ]

2 = Droughts [ ]

3 = Soil type [ ]

4 = Length of time matured cassava is left in ground [ ]

5 = Time of the year (e.g. wet or dry season) [ ]

6 = Other (specify):

1. If 3 = Yes (Q6). Describe the soil characteristics that bring about cassava bitterness. If soils are located in a certain location, mention this?

­­­­­­­­­­

|  |
| --- |
|  |
|  |

1. Which varieties are influenced by soil type?

|  |
| --- |
|  |
|  |

1. If 5 = Yes (Q6). Specify the season in which cassava root bitterness is observed?

|  |
| --- |
|  |
|  |

1. Which varieties are influenced by changes in season?

|  |
| --- |
|  |
|  |

1. Which varieties are influenced by the length of time matured cassava is left in the ground (plant age at harvest)?

|  |
| --- |
|  |
|  |

**Characteristics of soils on farmers’ fields and cassava cropping practices**

1. Local name for soil type of field? ……………………………………………………………………………
2. When was the cassava planted?

| **TIME PERIOD** | **Wet season** | | | | | **Dry season** | | | | | | | **Wet season** | | | | | **Dry season** | | | | |
| --- | --- | --- | --- | --- | --- | --- | --- | --- | --- | --- | --- | --- | --- | --- | --- | --- | --- | --- | --- | --- | --- | --- |
|  | **2011** | | | | | **2011** | | | | | | | **2012** | | | | | **2012** | | | | |
|  | **Dec** | **Jan** | **Feb** | **Mar** | **Apr** | **May** | **Jun** | **Jul** | **Aug** | **Sep** | **Oct** | **Nov** | **Dec** | **Jan** | **Feb** | **Mar** | **Apr** | **May** | **Jun** | **Jul** | **Aug** | **Sep** |
| **Tick month** | **W** |  |  |  |  |  |  |  |  |  |  |  |  |  |  |  |  |  |  |  |  |  |

| **TIME PERIOD** | **Wet season** | | | | | **Dry season** | | | | | | | **Wet season** | | | | | **Dry season** | | | | |
| --- | --- | --- | --- | --- | --- | --- | --- | --- | --- | --- | --- | --- | --- | --- | --- | --- | --- | --- | --- | --- | --- | --- |
|  | **2013** | | | | | **2013** | | | | | | | **2014** | | | | | **2014** | | | | |
|  | **Dec** | **Jan** | **Feb** | **Mar** | **Apr** | **May** | **Jun** | **Jul** | **Aug** | **Sep** | **Oct** | **Nov** | **Dec** | **Jan** | **Feb** | **Mar** | **Apr** | **May** | **Jun** | **Jul** | **Aug** | **Sep** |
| **Tick month** | **W** |  |  |  |  |  |  |  |  |  |  |  |  |  |  |  |  |  |  |  |  |  |

1. List down cassava varieties planted by the farmer this season and indicate the variety type (sweet or bitter) and whether they are improved or local.

| **Variety name** | 1. **Bitter/Sweet** | 1. **Local/Improved** |
| --- | --- | --- |
|  |  |  |
|  |  |  |
|  |  |  |
|  |  |  |
|  |  |  |
