# Supplementary material for: Farmers’ perceptions on the causes of cassava root bitterness: A case of konzo-affected Mtwara region, Tanzania
Source: PLoS One. 2019 Apr 18;14(4):e0215527. doi: 10.1371/journal.pone.0215527 (PMC6472768; doi:10.1371/journal.pone.0215527)
Supplement: S2 Text — (DOCX) [file pone.0215527.s002.docx]

**QUESTIONAIRE SYMBOL KEY**

**Some symbols**

888 = Not applicable; 999 = Blank/missing; 777 = I do not know; 1 = Yes; 0 = No

**Sources of income**

| Income 1 | Salary from formal employment |
| --- | --- |
| Income 2 | Sell of food surplus |
| Income 3 | Sell of cash crops |
| Income 4 | Hired labour |
| Income 5 | Income from cooperatives |
| Income 6 | Other income sources |
| Income 7 | AID |

**Surplus food crops**

| SurpCrop1 | Cassava |
| --- | --- |
| SurpCrop2 | Maize |
| SurpCrop3 | Pigeon pea |
| SurpCrop4 | Sorghum |
| SurpCrop5 | Groundnuts |
| SurpCrop6 | Bambara nuts |
| SurpCrop7 | Cowpeas |
| SurpCrop8 | Banana/plantain |
| SurpCrop9 | Millet |
| SurpCrop10 | Rice |

**Cash crops**

| CashCrop1 | Cashew |
| --- | --- |
| CashCrop2 | Sesame |
| CashCrop3 | Maize |
| CashCrop4 | Cowpeas |
| CashCrop5 | Bambara |
| CashCrop6 | Oranges |
| CashCrop7 | Pigeon peas |
| CashCrop8 | Groundnuts |
| CashCrop9 | Finger millet |
| CashCrop10 | Cassava |
| CashCrop11 | Coconut |
| CashCrop12 | Pineapple |

**Agronomic reasons**

| Agro1 | Type of variety |
| --- | --- |
| Agro2 | Droughts |
| Agro3 | Soil type |
| Agro4 | Length of time matured cassava is left in the ground |
| Agro5 | Time of the year (wet or dry season) |
| Agro6 | Piece meal harvesting |
| Agro7 | Stem cutting to enhance leaf production i.e. ratooning |
| Agro8 | Weeds |
| Agro10 | Semi-arid areas |

**Soil types causing bitterness**

1. Red soils/Red clayey soils
2. Over cultivated soil with poor soil fertility
3. Sandy soils

**Varieties that change taste with soil type**

1. Kigoma
2. All cassava varieties

NB: If variety type is not mentioned then it can be assumed that this happens to all varieties.

**Season in which bitter taste is observed** (label = BitternessSeason)

1. Dry season
2. Wet season

**Varieties that change taste with season**

| VarSeason1 | Mtukane |
| --- | --- |
| VarSeason2 | Mnalile kuchumba |
| VarSeason3 | Vincenti |
| VarSeason4 | Badi |
| VarSeason5 | Nachinyanya |
| VarSeason6 | Musa Saidi |
| VarSeason7 | Kigoma mafia |
| VarSeason8 | Liwoyoka |

**Varieties affected by length of time left in ground before harvest (plant age)**

| Varharv1 | Kigoma |
| --- | --- |
| Varharv2 | Kifuru |

**Varieties causing intoxication**

1. Nanjejeha
2. Chimaji
3. Mretete
4. Gologo
5. Limbanga
6. Salanga
7. Mohamed mfaume
8. Musa saidi
9. Namanjele
10. Namulunga
11. Lukombe
12. Mkwangulile
13. Mumdimbilile
14. Mpandinyeni

**Varieties planted**

| **Variety** | **Genotype** | **Type** |
| --- | --- | --- |
| 1. Nanjenjeha | Bitter | Local |
| 1. Salanga | Bitter | Local |
| 1. Limbanga | Bitter | Local |
| 1. Nakungóha | Bitter | Local |
| 1. Kigoma | Sweet | Local |
| 1. Naliendele | Sweet | Improved |
| 1. Chinanyanga | Sweet | Local |
| 1. Mapuya | Bitter | Local |
| 1. Nakulaha | Sweet | Local |
| 1. Sheria | Sweet | Local |
| 1. Lihumbuka | Sweet | Local |
| 1. Chemaji | Bitter | Local |
| 1. Mayunda | Sweet | Local |
| 1. Kifuru | Sweet | Local |
| 1. Salawende | Sweet | Local |
| 1. Mretete | Bitter | Local |
| 1. Mohamedi Mfaume | Bitter | Local |
| 1. Ng'amula mdukula | Sweet | Local |
| 1. Mtukane | Sweet | Local |
| 1. Badi | Sweet | Local |
| 1. Musa Saidi | Bitter | Local |
| 1. Mnalile Kuchumba | Sweet | Local |
| 1. Vincenti | Sweet | Local |
| 1. Nachinyanya | Sweet | Local |
| 1. Binti Juma | Sweet | Local |
| 1. Mpandinyeni | Bitter | Local |
| 1. Namanjele | Bitter | Local |
| 1. Msiliechunbani | Sweet | Local |
| 1. Albert | Sweet | Local |
| 1. Lukombe | Bitter | Local |
| 1. Liwoyoka | Sweet | Local |
| 1. Kigoma Mafia | Sweet | Local |
| 1. Mkwangulile | Bitter | Local |
| 1. Mumdimbilile | Bitter | Local |
| 1. Kiroba | Sweet | Improved |
| 1. Chihoba | Bitter | Local |
| 1. Mwendowaloya | Sweet | Local |
| 1. Kigoma Tanga | Sweet | Local |
| 1. Nakuchima | Bitter | Local |
| 1. Supa | Sweet | Local |
| 1. Likukulu | Bitter | Local |
| 1. Kifuu cha nazi | Sweet | Local |
| 1. Gologo | Sweet | Local |
| 1. Kigoma Maji | Sweet | Local |
| 1. Kigoma Baridi | Sweet | Local |
| 1. Kigomo Cheupe | Sweet | Local |

**Soil characteristics**

| **Soil texture** | **Colour** |
| --- | --- |
| 1. Loamy | 1. Red soil |
| 1. Sandy | 1. Grey soil |
| 1. Clayey | 1. Dark soil |
| 1. Sandy clay | 1. White soil |

**Planting time**

1. Early planted (November, December, January)
2. Mid-season (February, March)
3. Late season planted (April)
4. Off-season planted (September, October)
